# The control effect of orthokeratology on axial length elongation in Chinese children with myopia

**DOI:** 10.1186/1471-2415-14-141

**Published:** 2014-11-24

**Authors:** Meng-Jun Zhu, Hao-Yan Feng, Xian-Gui He, Hai-Dong Zou, Jian-Feng Zhu

**Affiliations:** Shanghai Eye Disease Prevention & Treatment Center, No. 380 Kangding Road, Jingan District, Shanghai, 200040 China

**Keywords:** Myopia, Orthokeratology, Axial length, Retrospective analysis

## Abstract

**Background:**

To retrospectively compare axial elongation in children with different degrees of myopia wearing spectacles and undergoing ortho-k treatment.

**Methods:**

The medical records of 128 patients who were fitted with spectacles or orthokeratology (ortho-k) lenses in our clinic between 2008 and 2009 were reviewed. Ortho-k group comprised 65 subjects and 63 subjects wearing spectacles were included in the control group. Subjects were also divided into low-myopia, moderate-myopia and high-myopia groups, based on the basic spherical equivalent refractive error. Axial length periodically measured over 2-year of lens wear and changes in axial length were compared between treatment groups and between subgroups with different degrees of myopia.

**Results:**

The control group exhibited more changes in axial length than the ortho-k group at both 12 months (0.39 ± 0.21 mm vs 0.16 ± 0.17 mm, p <0.001) and 24 months (0.70 ± 0.35 mm vs 0.34 ± 0.29 mm, p <0.001). Axial length elongation was estimated to be slower by about 51% in the ortho-k group. Similar results were found for the subgroups (49%, 59% and 46% reductions, respectively). In the group with low and moderate myopia, the annual increases in axial length were significantly different between the ortho-k and control groups during both the first ( Low myopia: 0.19 ± 0.17 mm vs 0.40 ± 0.18 mm, p = 0.001; Moderate myopia: 0.14 ± 0.18 mm vs 0.45 ± 0.22 mm, p <0.001) and second ( Low myopia: 0.18 ± 0.14 mm vs 0.32 ± 0.19 mm, p = 0.012; Moderate myopia: 0.18 ± 0.16 mm vs 0.34 ± 0.30 mm, p = 0.030) years. In the high myopia groups, significant differences were only found between the ortho-k and control groups during the first year (0.16 ± 0.18 mm vs 0.34 ± 0.22 mm, p = 0.004). The 2-year axial elongation was significantly associated with initial age (p <0.001) and treatment (p <0.001), but not with gender, initial refractive error, initial axial length, initial corneal curvature.

**Conclusions:**

This 2-year study indicates that ortho-k contact lens wear is effective for reducing myopia progression in children with low, moderate and high myopia.

## Background

Myopia is one of the most common ocular disorders and has become more prevalent in both adults and children [1, 2]. High myopia is associated with increased risks of retinal and vitreous detachments as well as other disorders, such as glaucoma and macular degeneration. High myopia is also associated with increased healthcare costs and ocular-related morbidity [3]. Therefore, many methods have been implemented to try to slow or stop the development of myopia in children [4]. These methods generally fall into two major categories: the topical application of tropicamide [5], atropine [6], pirenzepine [7] or some ocular hypotensive agent [8] or optical treatments, such as rigid contact lenses [9], bifocal spectacle lenses [10] or multifocal spectacle lenses [11]. However, none of these methods are ideal due to limitations in efficacy, safety, economic feasibility or ease of application.

Myopia is a remediable cause of visual impairment [12] and is one of the five priorities set by Vision 2020, the global initiative for the elimination of avoidable blindness, launched by the World Health Organization (WHO) [13]. Vision 2020 also seeks to increase awareness of the growing problem of myopia in children. Although the impact of myopia on quality of life is not as large as the impact from cataracts or other ocular pathologies [14], the age of onset for refractive errors suggests that this burden may be, in some ways, even greater than that of cataracts [15]. If effective treatment strategies can be found to reduce the rate of myopic progression, effects on socioeconomic health caused by myopia, can be profoundly reduced.

The specific mechanisms involved in the etiology of myopia are still unclear; however, there are currently three hypotheses regarding the progression of juvenile-onset myopia. First, it has been hypothesized that a high near-accommodation lag induces abnormal axial growth of the eye [16], though many studies have found no association between accommodative lag and myopic progression [17, 18]. The second hypothesis is based on longitudinal ocular growth data from emmetropic and myopic children and states that mechanical tension, created by the crystalline lens or ciliary body, restricts equatorial ocular expansion and causes accelerated axial elongation [19]. A thickened ciliary muscle [20] and thinned crystalline lens [21] play important roles in maintaining proportional expansion of the globe during eye growth and accelerated axial growth results [22] when the crystalline lens can no longer decrease in power by thinning and stretching [19]. The third hypothesis proposes that myopic eyes are relatively more hyperopic from the peripheral retina to the fovea so that when there are conflicting visual signals in the central and peripheral retina, the peripheral retinal signals dominate axial growth and central refractive development [22]. Longitudinal data, obtained two years prior to the onset of myopia in children who ultimately become myopic, has demonstrated a significant increase in relative peripheral hyperopia, and a relatively more prolate shape [19]. However, this hypothesis remains controversial [23]. Clinicians have been searching for treatments to control the progression of myopia based on these hypotheses; however, few studies have shown clinically significant results.

Orthokeratology (ortho-k) is defined as the clinical technique that uses specially designed and fitted rigid contact lenses to reshape the corneal contour to temporarily modify or eliminate refractive error. It was first introduced in the early 1960 s [24], but safety concerns and outcome unpredictability limited its use [25]. Since the mid-1990s, the acceptance of ortho-k has significantly increased, mainly due to huge technological developments in the field of contact lenses, such as the availability of highly-permeable materials [26] and the advent of advanced digital processing technology which uses computer assisted system to achieve optimal lens fitting [27]. A number of recent reports have demonstrated “overnight ortho-k” to be effective for controlling the progression of myopia [28–35]. In fact, several previous studies have reported the efficacy of ortho-k use in those with low to moderate myopia [28–32]; however, few studies have directly examined the effects of spherical, reverse geometry ortho-k lens designs on axial length elongation in highly myopic children [35, 36]. The purpose of the current study was to assess the effectiveness of ortho-k contact lenses in Chinese myopic children with different degrees of myopia over 24 months.

## Methods

### Subjects

The medical records of children who came to our clinic for vision correction using spectacles or ortho-k were reviewed and pertinent data was retrieved. A total of 128 files were included, 65 ortho-k treated subjects (ortho-k group) and 63 spectacle-wearing subjects (control group). Each group was designed to achieve 90% power to detect a minimum 0.18 mm (about 0.50D) difference in axial elongation in two years at the 5% level of statistical significance, using group standard deviation of 0.27 mm based on previous studies [28]. The minimal required sample size was calculated to be 49. Each group was further divided into three sub-groups based on the basic spherical equivalent refractive error (SER): low-myopia (−3.00D < SER < −0.50D), moderate-myopia (−6.00D < SER ≤ −3.0D) and high-myopia (SER ≤ −6.0D). The ortho-k group selection was based on the inclusion criteria listed in Table 1. The medical records of all ortho-k patients seen in our hospital from January 2008 to February 2009 were examined and those that met the inclusion criteria were sorted out. About twenty subjects were then randomly selected for inclusion in each sub-group, according to basic SER. For the control group, we chose all outpatient refractive files that matched inclusion criteria during the same time period as ortho-k patients; about 20 control subjects were randomly selected for each sub-group.

**Table 1:** **Inclusion criteria**

Age	Subjects were 7–14 years of age
Visual acuity	Had no other ocular diseases aside from refractive error and no keratoconus (confirmed by pre-treatment corneal topography)
Refractive errors	Had an intraocular pressure (IOP) of <21 mmHg
Ocular health	Had an with-the- rule astigmatism (axes 180 ± 30) ≤ 1.50 D
	Had a BCVA (best corrected visual acuity) ≤0.00 log MAR units in both eyes (Snellen equivalent to 20/20)
	Had no binocular vision problems
Others	No medications that might affect refractive development
	Had no history of ortho-k or contact lens wear
	Maintained regularly scheduled visits and completed the 2-year follow-up
	Had no significant deviations during lens wear (criteria only for ortho-k group)
	Discontinued lens wear a total of 30 days or less during the 2 years (criteria only for ortho-k group)

The current investigation followed the tenets of the Declaration of Helsinki and was approved by the Ethical Committee of the Shanghai Eye Disease Prevention and Treatment Center. All subjects, and their parents, provided signed written informed consent for treatment and were fully informed of the risks and benefits of the treatment.

### Materials

The lenses used in ortho-k group, Euclid overnight ortho-k lenses (Euclid Systems Corporation, Herndon, USA), were made of Boston EQUALENS II (oprifocon A) with an oxygen permeability coefficient of 85 × 10^−11^ (cm^2^ • ml0_2_) / (s•ml•mmHg), a diameter of 10.6 mm, an optical center thickness of 0.11-0.13 mm, a wetting angle of 36° and a four anti-arc inner surface geometry.

Appropriate trial lenses were chosen according to corneal curvature and other parameters (refractive error, corneal diameter). The subjects then tried the lenses for 30 minutes. Fluorescein pattern was used to observe the lens fit in each eye under slit lamp, before the final base and alignment curve of the lens was chosen. Ideal lenses had a good central position, a 0.5-1.0 mm range of movement, a 3.0-4.0 mm flat contact area in the center of the cornea, a 1.0-2.0 mm annulus of tear reservoir in the reverse curve, a parallel curve with the cornea and a 0.5-1.0 mm area of annulus of tear reservoir in the peripheral curve. Final lenses were determined after optimization and were worn overnight for ≥7 hours. The subjects were provided with clear instructions regarding the wearing and maintenance of lenses. Follow-up appointments were promptly planned (see Return visits).

Although the ortho-k lens design used in this study was recommended for myopia reduction not more than 6.00 D, we also used this design for subjects with high myopia. The main objective of ortho-k treatment was to slow myopic progression. If the subject’s refractive error was beyond the limit of correction range of ortho-k lens, a maximum manufacturer’s recommended diopter correction lens was prescribed under the corneal parameters condition. A subjective refraction was conducted in subjects, after 1 month of wearing the ortho-k lenses, to obtain the residual refraction error. Taking into account the further declining of myopia , pair of spectacles that we prescribed for subjects were 0.5D lower than the results of subjective refraction (residual refractive error greater than 0.50 D) to meet the needs of daily life of subjects temporary. A subjective refraction was again conducted after subjects had worn ortho-k lenses for 3 months and spectacles were prescribed according to the results of subjective refraction results. Thus, if full reduction in any degree of myopia was not achieved (residual refractive error greater than 0.50 D), the subjects were required to wear spectacles during the day. Spectacle prescription would be updated at any subsequent visit if diffierence in residual refractive error obtained at that visit exceeded 0.50D.

The control group wore single-vision spectacles. The lenses were modified according to visual acuity and refractive changes where appropriate throughout the study.

### Measurement techniques

Standard subjective refraction techniques were used to determine accurate refractive error. A pentacam (Oculus, Wetzlar, Germany) was used to monitor changes in corneal topography. Slit lamp biomicroscopy was used to monitor corneal health and integrity at each measurement session. Corneal integrity was assessed with topical application of sodium fluorescein dye. The axial length was evaluated using an IOLMaster (Carl Zeiss Jena GmbH, Jena, Germany). Three measurements were taken at each visit and the average of the 3 was used as a representative value.

### Return visits

For all subjects (both ortho-k and control groups), cycloplegic refraction was performed at the first visit. Non-cycloplegic refraction was also performed the following day. In the follow up visit, only non-cycloplegic subjective refraction were conducted. Subjects in the ortho-k group were examined 1, 7, 30 and 90 days after beginning ortho-k treatment. After the initial 90 days, subjects were examined every 3 months. Corneal integrity, corneal topography, visual acuity, refraction and IOP measurements were performed every 3 months and axial length was measured every 6 months. Subjects were also examined whenever an abnormal symptom occurred. If corneal staining was observed, ortho-k lens wear were asked to be ceased for 2 days for low level staining (grade 1, Efron Grading Scale), and 5 ~ 7 days for more intense staining (grade 2 and grade 3, Efron Grading Scale) first.Then subjects should return for examination following doctor’s orders and resumed lens wear until their corneal were completed recovered.Data collection was resumed after a few days resuming lens wear as necessary. All examinations were performed within 2 hours of removing the lens (between 8 and 10 o’clock in the morning). In the control group, axial length, refraction and visual acuity were measured and these examinations were performed every 6 months until the end of the 2-year study period.

### Data analysis

Since all data were distributed normally (Kolmogorov-Smirnov test, P >0.05), parametric tests were used for data analysis. Unpaired t tests were used to compare the baseline data and Chi-square tests were used to compare the difference in the male/female ratio (M/F ratio) between the two groups of subjects. To determine myopic progression, repeated measures analysis of variance (ANOVAs) were used to compare the change in axial length over time in the two groups. If significant differences were found, paired *t* tests with Bonferroni correction were performed to compare the differences in axial length. An unpaired *t*-test was used to compare the annual increases in axial length between the two study groups during the 2-year treatment period. Factors affecting axial elongation, including age, gender, treatment, initial spherical equivalent refractive error and initial corneal curvature were examined using stepwise multiple linear regression analysis. Data analysis was performed in SPSS statistical software program (SPSS software ver. 16.0; SPSS Inc., Chicago, IL, USA). The level of statistical significance was set at 5%. Only data from the right eyes of the subjects were analyzed.

## Results

Ortho-k group comprised 65 subjects (23 boys and 42 girls), ranging in age from 7 to 14 years. The SER ranged from −1.25 to −2.75 D in the low myopia group, from −3.25 to −5.50 D in the moderate myopia group and from −6.00 to −7.88 D in the high myopia group (Table 2).

**Table 2:** **Baseline demographics and biometric data of subjects enrolled in the study**

	Ortho-k	Control	***P*** value
Total	N = 65	N = 63	
Age (y)	9.82 ± 1.63	9.86 ± 2.12	0.923*
M/F ratio	23:42	26:37	0.134†
SER (D)	−4.29 ± 2.04	−4.24 ± 2.38	0.898*
Corneal curvature (D)	43.45 ± 1.27	43.45 ± 1.47	0.999*
Axial length (mm)	24.91 ± 0.83	24.85 ± 1.08	0.725*
Low myopia	N = 20	N = 20	
Age (y)	9.23 ± 1.53	8.87 ± 1.71	0.481*
M/F ratio	7:13	8:12	0.744†
SER (D)	−1.99 ± 0.56	−1.64 ± 0.62	0.074*
Corneal curvature (D)	43.07 ± 1.54	43.41 ± 1.71	0.521*
Axial length (mm)	24.29 ± 0.73	23.97 ± 0.87	0.207*
Moderate myopia	N = 23	N = 21	
Age (y)	9.72 ± 1.22	10.16 ± 1.82	0.358*
M/F ratio	9:14	9:12	0.802†
SER (D)	−3.97 ± 0.77	−3.82 ± 0.53	0.439*
Corneal curvature (D)	43.45 ± 1.02	43.56 ± 1.41	0.765*
Axial length (mm)	24.78 ± 0.40	24.74 ± 0.83	0.829*
High myopia	N = 22	N = 22	
Age (y)	10.48 ± 1.92	10.47 ± 2.46	0.995*
M/F ratio	7:15	9:13	0.531†
SER (D)	−6.72 ± 0.62	−7.01 ± 1.18	0.316*
Corneal curvature (D)	43.79 ± 1.19	43.39 ± 1.33	0.290*
Axial length (mm)	25.61 ± 0.73	25.77 ± 0.70	0.477*

*Unpaired *t*-test.

†Chi-square test.

Spectacle control group comprised 63 subjects (26 boys and 37 girls), ranging in age from 7 to 14 years. In this group, the SER ranged from −1.00 to −2.75 D in the low myopia control group, from −3.13 to −4.75 D in moderate myopia control group and from −6.00 to −10.13 D in the high myopia control group (Table 2).

There were no statistically significant differences in baseline values between the ortho-k and spectacle control groups in terms of age, gender distribution, SER, corneal curvature, axial length (*P* >0.05, unpaired *t*-test and Chi-square test, Table 2). Baseline characteristics of the different degrees of myopia were also balanced between the two groups (Table 2).

**Figure 1:**
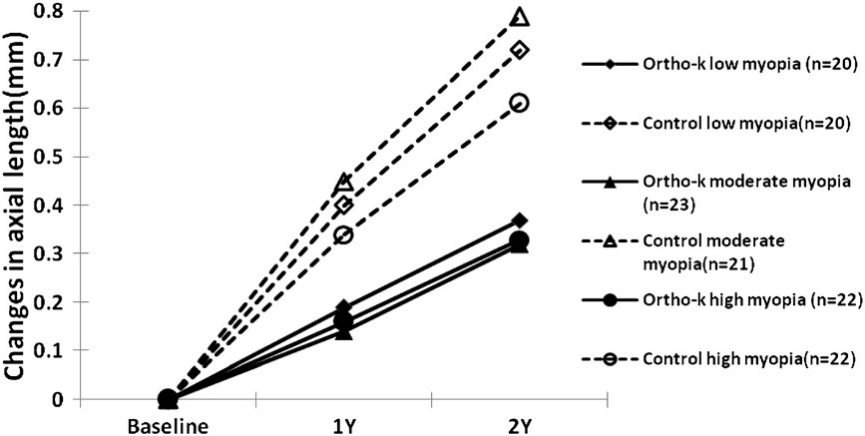
**Axial elongation (mm) over the 2-year treatment period in subjects with different degrees of myopia in the ortho-k and spectacle groups.**

The mean (±SD) SER and axial length of the Ortho-k and spectacle subgroups are listed in Table 3. The SER changed significantly after 2-year treatment in different degree of myopia for both group (p <0.001, repeated-measures ANOVA). There was also a significant elongation in axial length in both the ortho-k and spectacle control groups over the time course of the study (p < 0.001, repeated-measures ANOVA). During the 2 year period, the spectacle control group exhibited more change in axial length than did the ortho-k group during both the first year (0.39 ± 0.21 mm vs 0.16 ± 0.17 mm,; 59% slower in the ortho-k group) and the second year (0.31 ± 0.24 mm vs 0.18 ± 0.15 mm,; 42% slower in the ortho-k group). In total, the increase in axial elongation was 51% slower in the ortho-k group (0.70 ± 0.35 mm in control group vs 0.34 ± 0.29 mm in ortho-k group) at the end of the 24-month study period. Similar results were found in the subgroups. There was a significant difference in axial elongation when comparing ortho-k and control groups with low and moderate myopia during the first year and the second year. When comparing the ortho-k and spectacle control groups with high myopia, the difference in axial elongation was only found during the first year, but not during the second year. At the end of the 2-year study, significant differences in total axial elongation were found for all degrees of myopia. The elongation of axial length was 49%, 59%, and 46% slower for the low, moderate and high myopia subgroups, respectively (53%, 69%, and 53% slower during the first year and 44%, 47%, and 33% slower during the second year) (Figure 1, Table 4).

**Table 3:** **Mean (±SD) spherical equivalent refractive error and axial length in different degree of myopia for the ortho-k and spectacle groups during the study period**

	1 year	2 year
Axial length		
Low myopia		
Ortho-k	24.48 ± 0.75	24.67 ± 0.74
Control	24.36 ± 0.88	24.69 ± 0.81
Moderate myopia		
Ortho-k	24.92 ± 0.35	25.11 ± 0.37
Control	25.19 ± 0.79	25.53 ± 0.76
High myopia		
Ortho-k	25.77 ± 0.78	25.95 ± 0.89
Control	26.11 ± 0.70	26.38 ± 0.70
Total		
Ortho-k	25.07 ± 0.82	25.26 ± 0.87
Control	25.25 ± 1.06	25.56 ± 1.02
spherical equivalent refractive error (SER)		
Low myopia		
Ortho-k	0.00 ± 0.24	−0.22 ± 0.24
Control	−2.09 ± 0.43	−2.73 ± 0.77
Moderate myopia		
Ortho-k	−0.36 ± 0.45	−0.71 ± 0.45
Control	−4.52 ± 0.86	−5.06 ± 0.98
High myopia		
Ortho-k	−1.97 ± 1.30	−2.17 ± 1.30
Control	−7.59 ± 1.34	−8.19 ± 1.39
Total		
Ortho-k	−0.80 ± 1.18	−1.05 ± 1.15
Control	−5.02 ± 2.34	−5.60 ± 2.38

**Table 4:** **Changes in axial length during the 2-year treatment of subjects with different degrees of myopia between the ortho-k group (65 subjects) and spectacle group (63 subjects)**

	First year	Second year	2 years
Low myopia			
Ortho-k	0.19 ± 0.17	0.18 ± 0.14	0.37 ± 0.28
Control	0.40 ± 0.18	0.32 ± 0.19	0.72 ± 0.28
t	−3.80	−2.64	−3.97
P	0.001	0.012	<0.001
Moderate myopia			
Ortho-k	0.14 ± 0.18	0.18 ± 0.16	0.32 ± 0.31
Control	0.45 ± 0.22	0.34 ± 0.30	0.79 ± 0.39
t	−5.09	−2.24	−4.40
P	<0.001	0.030	<0.001
High myopia			
Ortho-k	0.16 ± 0.18	0.18 ± 0.15	0.33 ± 0.30
Control	0.34 ± 0.22	0.27 ± 0.21	0.61 ± 0.36
t	−3.05	−1.651	−2.814
P	0.004	0.107	0.007
Total			
Ortho-k	0.16 ± 0.17	0.18 ± 0.15	0.34 ± 0.29
Control	0.39 ± 0.21	0.31 ± 0.24	0.70 ± 0.35
t	−6.87	−3.699	−6.408
P	<0.001	<0.001	<0.001

**Figure 2:**
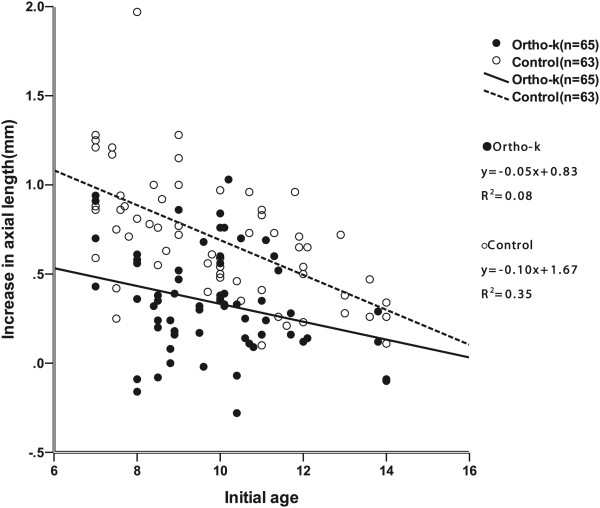
**Scatterplots showing correlations of axial growth at the 24-month visit with baseline age in ortho-k and control groups.**

**Figure 3:**
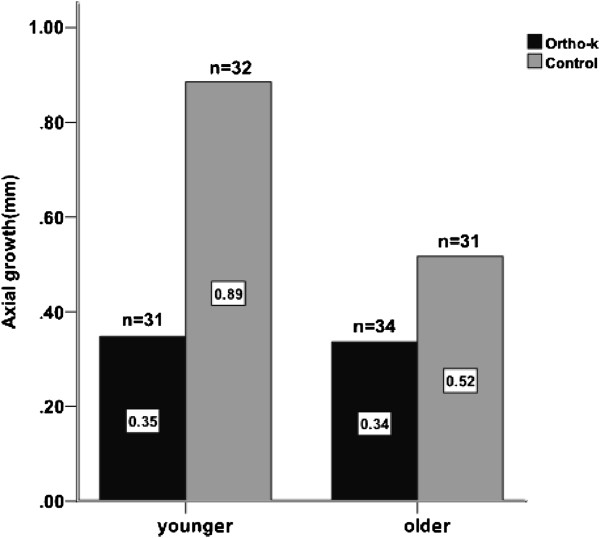
**Changes in axial length in younger (age below average) and older (age above average) children in the ortho-k and control groups after 2-year monitoring.**

The regression of the model using treatment and initial age to predict axial elongation was fair (adjusted *R*^*2*^ = 0.41) and significant (p <0.001). Hence, linear regression of axial elongation and initial age was performed for each group. Figure 2 shows the axial elongation plotted against the initial ages during the 2-year period for both groups. The linear regression equation is y = −0.05x + 0.83 (*R*^*2*^ = 0.08, p = 0.023) for the ortho-k group and y = −0.10x + 1.67 (*R*^*2*^ = 0.35, p < 0.001) for the control group.The mean age of all subjects (9.84 ± 1.88 years) was selected as the cutoff value and subjects were divided into two age groups. Subjects ranging in age from 7 to 9.8 years were considered to be the younger group, whereas subjects older than 9.9 years of age were considered to be the older group. As shown in Figure 3, axial elongation was nearly 61% slower in the ortho-k subjects compared with the control subjects in the younger group after 2 years. In the older group, axial elongation was only 35% slower in ortho-k versus control subjects.

During the 2-year study period, corneal staining (grade 1 ~ grade 3, Efron Grading Scale) was found in 17 ortho-k subjects. Through clinical examination, all subjects completely recovered after discontinuation of lens wear for 2–14 days. There were no adverse events reported in the spectacle group.

## Discussion

The data from the current study support the theory that ortho-k can reduce progression of myopia by approximately half compared with traditional spectacle lenses. The major strength of the current study is the inclusion of a greater number of subjects with moderate and high myopia compared to previous studies [30–32], which allowed the effectiveness of ortho-k lens to be evaluated in those with different degrees of myopia.

Cho *et al.*[28] first reported that an increase in axial length of 0.29 mm in an ortho-k-treated group and of 0.54 mm in a spectacle group over a 2-year period. Since then, similar results have been obtained in several other studies [29–33]. Walline *et al.*[30] reported that the increase in axial length after 2 years was 0.25 mm in an ortho-k group and 0.57 mm in a control group. Kakita *et al.*[29] and Santodomingo-Rubido *et al.*[32] also reported differences in axial length increases between myopic children wearing ortho-k contact lenses and those wearing single-vision spectacles (0.39 mm vs 0.61 mm and 0.47 mm vs 0.69 mm, respectively), over a 2-year period. In a randomised study, Cho and Cheung [34] reported slower axial elongation of 43% in low myopes. Chen *et al.*[33] reported 52% slower increase in axial length with toric ortho-k. The change in axial length growth between the ortho-k and spectacle groups found in our study is reasonably consistent with previously reported studies, although variations in ethnicity, age and basic refractive error between studies likely affect the rates of myopic progression [28–34].

The prevalence of high myopia (greater than −5.00 D) among Chinese adults (greater than 30 years of age) has been reported to be 2% to 5% [37, 38], whereas the prevalence of high myopia (greater than −6.00 D) among Chinese children, aged 5 to 16, has been reported to be approximately 1.19% [39]. Pathologic complications, poor vision quality and decreased quality of life are all associated with high myopia. Controlling the rate of myopic progression, thereby reducing the prevalence of high myopia, may result in fewer pathologic complications and improved quality of life [40]. Since children with severe myopia have faster myopic progression [39] in general, it is logical that they would benefit most from a treatment that retards myopic progression. However, information about treatments for high myopia is rare since most studies examining treatments for myopia had been performed in children with low to moderate myopia. In a small study, involving 20 highly myopic children (greater than −6.00 D), myopic progression was found to be significantly reduced after treatment with 0.5% atropine eye drops [41]. A subsequent case–control study indicated that 1% topical atropine was effective in slowing myopic progression in moderately to severely myopic children (initial refractive errors: −5.18 ± 2.05 D) at the end of 1 year of treatment [42]. Chen, Cheung and Cho [43] reported no significant increase in axial length of two highly myopic, astigmatic subjects, with histories of myopic progression after they were treated with toric, ortho-k lenses. A recent randomized study showed that ortho-k lenses effectively slowed myopic progression in high myopes. In that study, which axial length elongation was reported to be 63% slower in children treated with partial correction ortho-k lenses compared to children wearing spectacles [35]. In the current study axial length elongation was found to be 46% slower in the high myopic ortho-k group compared with the spectacle group. Although full reduction was not achieved in the current study with current ortho-k lens designs, ortho-k was shown to be effective to slow myopic progression during the 24-month period of lens wear in subjects with low, moderate and high myopia. Previous studies have reported that subjects with higher baseline SERs benefited the most from ortho-k treatment [29, 30]. However, in this study, subjects with all degrees of myopia demonstrated similar therapeutic benefits from ortho-k, specifically in retarding axial growth.

The results of the current study are in agreement with previous studies in that there is a significant negative correlation between initial age and the change in axial length after 24 months of ortho-k treatment. This result is supported by observations in 3 other recent studies [31, 33, 34]. Hence, we concurred with Cho and Cheung [34] that younger myopic children will benefit more from ortho-k treatment than older myopic children. The annual axial elongation in the current study in high myopia group was 0.16 and 0.18 mm in the first and second years, respectively, in the ortho-k subjects, and was 0.34 and 0.27 mm, respectively, in the control subjects. Our results had little difference with the annual growth in the first year as reported by Charm J and Cho P [35], whose results showed relatively better myopic control in the first year of the study period (80% slower) compared with the second year (38% slower), while the value in current study was only 53% in the first year and 33% in the second year. This difference may be due to the different inclusion criteria in age of the two studies (8–11 years of age in Charm J and Cho P’s study and 7–14 years of age in our study). But the tendency of reduced myopic control effect in high myopia was same and this phenomena was also occurred in low and moderate myopia in current study. This may be due to the slowing of myopic progression in the control group [31, 35]. Another explanation may be the adaptation of subjects to the signal that slows myopic progression in the ortho-k group [34]. However, information on the effectiveness of ortho-k was only available over a 2-year period. It remains to be seen whether ortho-k lenses should be worn continually, what treatment duration will optimize the reduction in myopic progression. Further studies are needed to address these questions.

One limitation of this study is that this was a retrospective study. Factors may affect myopic progression, such as peripheral refractive status, accommodative lag, pupil size, retinal image quality, a history of parental myopia, were not recorded in either group. A second potential limitation is that, although there were already some articles about the short-term changes in ocular biometry after discontinuation of orthokeratology [44–46], it is unknown whether the rate of axial elongation will be maintained after cessation of ortho-k treatment [47] or whether a rebound phenomenon will occur as reported in the atropine study [48]. Further studies, including a longer follow-up period after cessation of treatment are required to answer these questions (especially in high myopia). Third, only one ortho-k lens spherical design was used in our study. There are a number of different lenses aimed at controlling myopia in moderate to high astigmatic and high myopic children [43], such as the toric reverse geometry lens, which provides good lens centration on toric corneas. Further investigation is warranted to address the long-term safety and myopia control efficacy in these specially designed ortho-k lenses.

## Conclusion

In conclusion, ortho-k contact lens wear is a promising strategy for reducing myopic development in myopic children. Elongation of axial length compared with subjects wearing spectacles was slower by 49%, 59% and 46% for low, moderate and high myopia during 2-year period. Ortho-k treatment would be more beneficial to younger myopic children. Early initiation of ortho-k treatment may be possible to reduce the prevalence of high myopia.

## Abbreviations

Ortho-K: Orthokeratology
IOP: Intraocular pressure
BCVA: Best corrected visual acuity
AL: Axial length
SER: Spherical equivalent refractive error.
